# The Role of the Clinical Pharmacist in Hospital Admission Medication Reconciliation in Low-Resource Settings

**DOI:** 10.3390/pharmacy13040107

**Published:** 2025-08-02

**Authors:** Tijana Kovačević, Sonja Nedinić, Vedrana Barišić, Branislava Miljković, Emir Fazlić, Slobodan Vukadinović, Pedja Kovačević

**Affiliations:** 1 Pharmacy Department, University Clinical Centre of the Republic of Srpska, Dvanaest Beba bb, 78000 Banja Luka, Bosnia and Herzegovina; sonja.nedinic@kc-bl.com (S.N.); vedrana.barisic@kc-bl.com (V.B.); 2 Faculty of Medicine, University of Banja Luka, Save Mrkalja 14, 78000 Banja Luka, Bosnia and Herzegovina; 3 Faculty of Pharmacy, University of Belgrade, Vojvode Stepe 450, 11000 Belgrade, Serbia; branislava.miljkovic@pharmacy.bg.ac.rs; 4 Faculty of Pharmacy, University of Sarajevo, 71000 Sarajevo, Bosnia and Herzegovina; emir.fazlic@medis.ba; 5 Abdominal Surgery Department, University Clinical Centre of the Republic of Srpska, Dvanaest Beba bb, 78000 Banja Luka, Bosnia and Herzegovina; slobodan.vukadinovic@kc-bl.com; 6 Medical Intensive Care Unit, University Clinical Centre of the Republic of Srpska, Dvanaest Beba bb, 78000 Banja Luka, Bosnia and Herzegovina

**Keywords:** medication errors, pharmacist, clinical, hospital admission, medication reconciliation

## Abstract

Medication discrepancies at hospital admission are common and may lead to adverse outcomes. Medication reconciliation is a critical process for minimizing medication discrepancies and medication errors at the time of hospital admission. This study aimed to evaluate the role of clinical pharmacists in identifying pharmacotherapy-related issues upon patient admission in a low-resource setting. A prospective observational study was conducted at a university hospital between 1 March and 31 May 2023. Within 24 h of admission, a clinical pharmacist documented each patient’s pre-admission medication regimen and compared it with the medication history obtained by the admitting physician. Discrepancies and pharmacotherapy problems were subsequently identified. Among 65 patients, pharmacists documented 334 medications versus 189 recorded by physicians (*p* < 0.01). The clinical pharmacist identified 155 discrepancies, 112 (72.26%) of which were unintentional. The most frequent type was drug omission (91.07%), followed by incorrect dosage (4.46%), incorrect dosing interval (2.68%), and medications with unknown indications (1.79%). Most discrepancies were classified as errors without harm (53.57%), while 41.07% were potentially harmful. These findings underscore the importance of integrating clinical pharmacists into the healthcare team. Their active participation during hospital admission can significantly enhance medication safety and reduce preventable adverse drug events.

## 1. Introduction

Medication discrepancies (MDs) are defined as any observed disagreement in the way medications are used at different levels of healthcare. These discrepancies are typically classified as intentional or unintentional, and further subcategorized as documented or undocumented. Intentional and clinically justified discrepancies that are properly documented are not considered errors; however, undocumented intentional discrepancies are recognized as potential pharmacotherapy problems [1]. Among all types, medication omissions are the most common and may significantly affect the course of a patient’s treatment. Such omissions are considered pharmacotherapy errors and can lead to the manifestation of side effects. It is estimated that up to 40% of pharmacotherapy discrepancies occur during hospital admission, intra-hospital transfers, or at discharge [2].

The Institute for Healthcare Improvement (IHI) defines the medication reconciliation process as the systematic approach to obtaining accurate and complete information on all medications a patient is currently taking—including the name, dosage, frequency, and route of administration—and comparing this information with the medication orders documented at key transition points, such as admission, transfer, and discharge. The primary goal of medication reconciliation is to ensure the safe and appropriate use of medications throughout the continuum of care during hospitalization [3].

Accurate documentation of all medications upon hospital admission is essential for determining appropriate dosing regimens throughout hospitalization. A complete and precise medication list contributes to the safe use of pharmacotherapy and helps prevent various types of medication errors, ultimately reducing the overall cost of treatment. Studies have reported that the prevalence of medication errors identified at admission ranges from 26.9% to 86.8%, highlighting the importance of medication reconciliation at the time of admission and during intra-hospital transfers as a key measure in preventing and minimizing adverse drug reactions (ADRs) [4,5]. An incomplete or inaccurate medication history upon admission may lead to MDs or inappropriate therapy during hospitalization, potentially compromising patient safety and clinical outcomes [6]. One study found that 37% of patients had at least one MD at admission, with the most frequent types being omitted medication (35%), incorrect dose (35%), and improper dosing interval (27%) [7].

**Figure 1:**
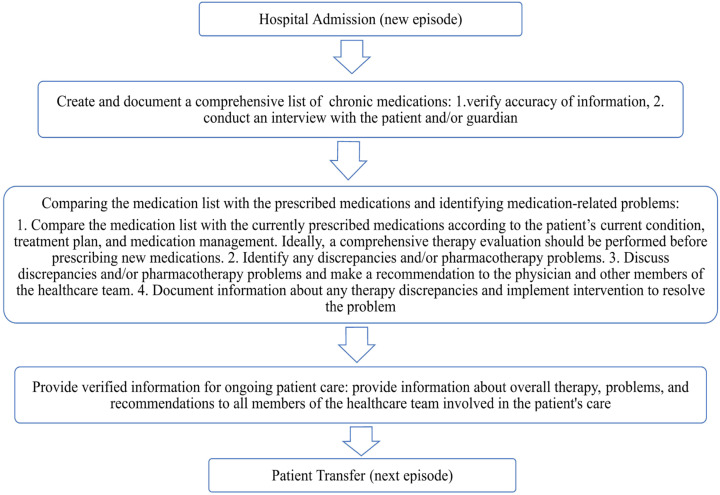
Medication reconciliation process.

According to recommendations from international organizations, hospitals should implement a structured medication reconciliation process upon admission to reduce the risk of unintentional medication errors and enhance patient safety [8]. The primary goal of medication reconciliation is to ensure that patients continue receiving all necessary medications as intended, while avoiding prescription errors, omissions, therapeutic duplications, and harmful drug–drug or drug–disease interactions [9]. This process should be standardized and initiated as early as possible upon admission, preferably within the first 24 h. Furthermore, all modifications to therapy should be clearly documented, and any discrepancies between the medication history obtained at admission and the current therapeutic plan must be recorded and addressed accordingly (Figure 1) [10].

Observed discrepancies in medication lists can be classified as unintentional, intentional, and undocumented intentional deviations. It is essential to communicate with the prescribing physician to determine whether the identified discrepancies are intentional or unintentional and to make the necessary therapeutic adjustments accordingly [11].

Special attention should be given to vulnerable patient populations during the medication history-taking process, including older adults, individuals with multiple comorbidities, and patients receiving multiple medications (polypharmacy). Although no universally accepted definition exists, polypharmacy is commonly defined as the concurrent use of five or more medications. Older patients are particularly at risk due to the increased likelihood of drug interactions, ADRs, cognitive impairment, and reduced adherence. However, regardless of age, an increase in the number of medications is associated with a higher likelihood of discrepancies in the medication history [8]. Other factors that may contribute to discrepancies include the use of high-risk medications, the timing of hospital admission (e.g., weekends or nights), and the type of hospital admission (emergency vs. elective) [12]. High-risk medications are defined as drugs that bear a heightened risk of causing significant harm when used incorrectly [13].

A systematic review of nineteen studies found that 20% of hospital readmissions are medication-related, and 69% of these readmissions are potentially preventable. The most common risk factors include older age, the presence of multiple comorbidities, and polypharmacy [14]. To reduce the rate of hospital readmissions, it is essential to accurately identify the underlying causes in order to implement the most effective interventions. This process requires gathering comprehensive information from the patient, including details on the method of medication administration, all regularly used medications—whether prescription or over-the-counter—as well as a history of any previous ADRs [15,16].

By carefully evaluating the benefits and risks of each medication, screening for drug–drug interactions, and continuously monitoring therapeutic outcomes, ADRs can be both prevented and promptly recognized. Early identification of ADRs allows for timely interventions that minimize potential harm. However, if an ADR is misinterpreted as a new medical condition requiring treatment (treating a side effect of an already administered drug), it may result in the prescription of an additional drug—a practice known as cascade prescribing. This can lead to unnecessary pharmacotherapy, further increasing the risk of adverse effects and drug interactions, while also contributing to higher healthcare costs and potential patient harm [17].

The National Coordinating Council for Medication Error Reporting and Prevention (NCC MERP) has categorized medication errors according to severity into four groups, or nine categories, in the Pharmacotherapeutic Error Categorization Index, as shown in Table 1 [18].

Identification of treatment-related problems is essential for achieving therapeutic goals and is one of the key roles of the clinical pharmacist. Recognizing MDs helps prevent adverse outcomes, which is crucial not only for patient safety but also for the efficiency of the healthcare system as a whole [16].

According to the European Society of Clinical Pharmacy (ESCP), clinical pharmacists, as integral members of the healthcare team, are responsible for ensuring the safe and appropriate use of medications. During hospitalization, clinical pharmacists carry out several key activities to identify MDs, including:

- Medication reconciliation
- Medication management
- Therapeutic drug monitoring (TDM)
- Patient education at discharge [19].

The involvement of a clinical pharmacist during care transitions ensures proper medication use, provides verified medication information to all members of the healthcare team, and facilitates the monitoring of patients at high risk for ADRs. Pharmacotherapy reviews conducted by clinical pharmacists during these transitions reduce the likelihood of therapy-related adverse outcomes and subsequent hospital readmissions [10].

Incomplete medication histories are commonly recorded by physicians during hospital admission, which may result in medication omissions and compromise patient outcomes. Clinical pharmacist involvement in medication coordination during hospitalization has been shown to significantly reduce the rate of unintentional MDs [20].

Numerous studies have demonstrated that pharmacists, owing to their comprehensive pharmacological knowledge and communication skills, are best equipped to gather accurate and complete medication histories at admission. Their collaboration with physicians and other healthcare professionals, combined with their consistency and dedication, plays a critical role in identifying MDs [21,22].

Most of these studies were conducted in high-resource settings where hospitals have established patient safety systems [23]. However, over two-thirds of the global population reside in countries identified as low-resource settings where progress in developing clinical pharmacy is hampered by various barriers, including the absence of clear policies, outdated academic curricula, workforce shortages, lack of effective incentive structures, and limited opportunities for practical training. Further challenges—such as insufficient clinical and communication skills, low self-confidence, negative professional attitudes, and the longstanding dominance of physicians in healthcare—also contribute to the slow pace of development [24]. The aim of this study was to evaluate the impact of clinical pharmacist-led medication reconciliation on hospital admission in a low-resource setting.

## 2. Materials and Methods

### 2.1. Study Design and Settings

This prospective observational study was conducted at the University Clinical Center of the Republic of Srpska (UCC RS), specifically within the abdominal surgery, orthopedics, and internal medicine wards (gastroenterology and endocrinology). UCC RS is a 1200-bed teaching hospital which comprises 27 wards and serves as a regional medical center for the Republic of Srpska.

The study was approved by the Ethics Committee of the UCC RS (No. 01-19-45-2/21).

### 2.2. Patients and Methods

Between 1 March and 31 May 2023, a prospective observational analysis of hospitalized patients’ pharmacotherapy was performed. The study included adult patients admitted to the selected wards who had been taking at least one chronic medication prior to admission.

Pharmacists collected comprehensive data on patients’ medication use within 24 h of admission. This information was then compared to the medication history recorded by the attending physician to identify MDs. Patients who were not taking any medications prior to admission or who were transferred from other hospital departments were excluded.

Data sources used to compile the complete pre-admission medication list included patient or caregiver interviews, primary care (family medicine) records, previous medical documentation, and medications brought by patients upon hospital entry. For patients undergoing planned interventions, anesthesiologist reports were also reviewed.

A structured questionnaire was used to collect the following information:

1. All pre-admission medications (name, dosage, pharmaceutical form, route of administration, dosing interval, duration, and indication);
2. Over-the-counter (OTC) medications, herbal products, and dietary supplements;
3. History of drug allergies and previously experienced ADRs;
4. Medication adherence.

Once the medication list was obtained, it was compared with the physician’s record in the clinical information system. Pharmacists discussed any identified discrepancies with the attending physicians to determine whether they were intentional or unintentional.

In addition, the following data were collected: patient initials, age, sex, lifestyle habits, method of medication organization at home, type of hospital admission (emergency or planned), admission diagnosis, comorbidities, and laboratory values. Information was retrieved from the hospital chart, family medicine records, anesthesia documentation, laboratory findings, and other available medical documentation.

### 2.3. Outcomes

The primary outcome was the difference in the number of medications recorded by pharmacists versus those documented by physicians upon admission. The secondary outcome was the classification of unintentional discrepancies in the medication histories and an assessment of their potential clinical impact using the NCC MERP Index for Categorization of Pharmacotherapeutic Problems.

### 2.4. Statistical Analysis

Descriptive and comparative statistical methods were used for data analysis. Continuous variables were presented as means with standard deviations or medians with interquartile ranges, while categorical variables were reported as numbers and percentages. Normality was assessed using the Kolmogorov–Smirnov and Shapiro–Wilk tests. Comparisons between continuous variables were performed using Student’s *t*-test or Mann–Whitney *U*-test, and categorical variables were compared using the Pearson’s chi-square test or Fisher’s exact test, as appropriate. A *p*-value of <0.05 was considered statistically significant. All statistical analyses were performed using SPSS version 26 [25].

## 3. Results

Medication data were collected for a total of 65 patients admitted to the abdominal surgery, orthopedic surgery, and internal medicine wards. Data collection was performed within 24 h of hospital admission. The demographic characteristics of the study participants are presented in Table 2.

Medication reconciliation performed by the clinical pharmacist upon hospital admission identified a total of 334 medications, whereas the physicians recorded only 189 medications during the initial anamnesis. A statistically significant difference was observed between the median number of medications recorded by pharmacists and physicians (*p* < 0.01), as presented in Table 3.

**Figure 2:**
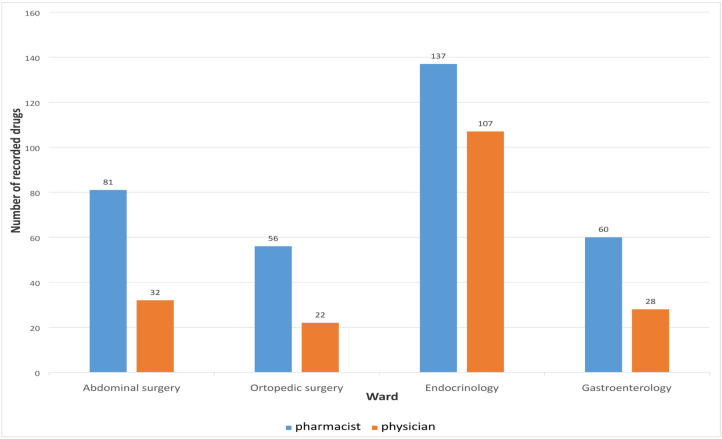
Number of medications recorded on different wards.

The number of medications recorded by physicians and clinical pharmacists upon admission, across different hospital wards, is presented in Figure 2.

**Figure 3:**
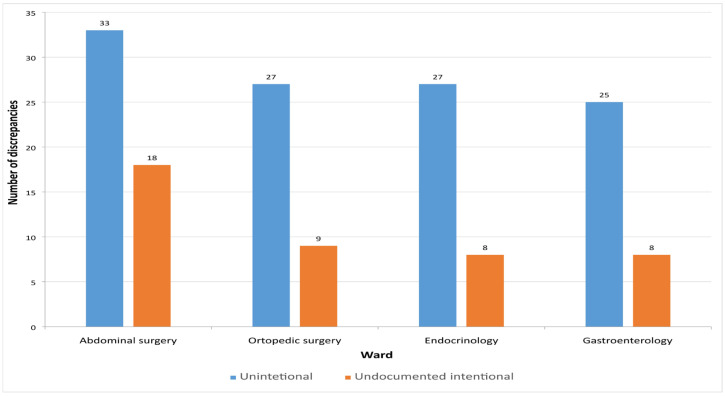
Number of detected discrepancies on admission across the hospital wards.

In this study, a total of 155 MDs were identified. Among them, 112 (72.26%) were unintentional, representing incomplete or inaccurate entries on the medication list, while 43 (27.74%) were intentional but undocumented discrepancies that were clinically justified. Figure 3 presents the distribution of unintentional and undocumented intentional discrepancies across the different hospital departments.

Further data analysis included only unintentional discrepancies.

The highest percentage of unintentional discrepancies during admission was related to omitted medications 102 (91.07%). Other unintentional discrepancies were represented in a small percentage and were related to incorrect dosage 5 (4.46%), dosage interval 3 (2.68%), and medication without indication 2 (1.79%).

**Figure 4:**
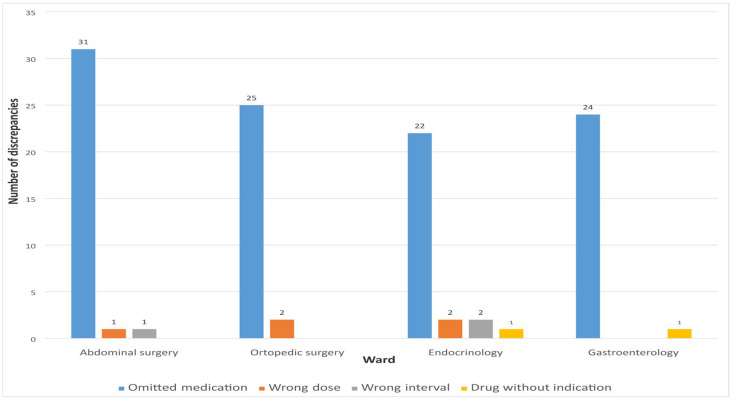
Types of detected discrepancies on admission.

Figure 4 shows the number of different types of MDs observed when comparing the list of medications recorded by clinical pharmacists and physicians during hospital admission.

A statistically significant (*p* < 0.05) association between patient age and the number of MDs was observed, as shown in Table 4.

Table 5 presents the difference in the number of MDs between urgent and scheduled hospital admissions, where the median number of discrepancies was higher in patients admitted urgently; however, the difference was not statistically significant.

According to the ATC classification, the drug group most frequently involved in unintentional MDs was group C (drugs that effect the cardiovascular system), followed by group A (drugs that affect the alimentary tract and metabolism) and group N (drugs that affect the nervous system). Table 6 presents the number of drugs associated with MDs categorized by ATC classification.

The assessment of the potential severity of MDs was conducted exclusively for unintentional discrepancies using the NCC MERP Index for Categorizing Medication Errors. Of the total 112 unintentional discrepancies, 60 (53.57%) were classified as errors without harm, while 46 (41.07%) were categorized as errors with potential to cause harm. Six discrepancies (5.36%) were not considered errors. No discrepancies were identified that could potentially result in fatal outcomes.

**Figure 5:**
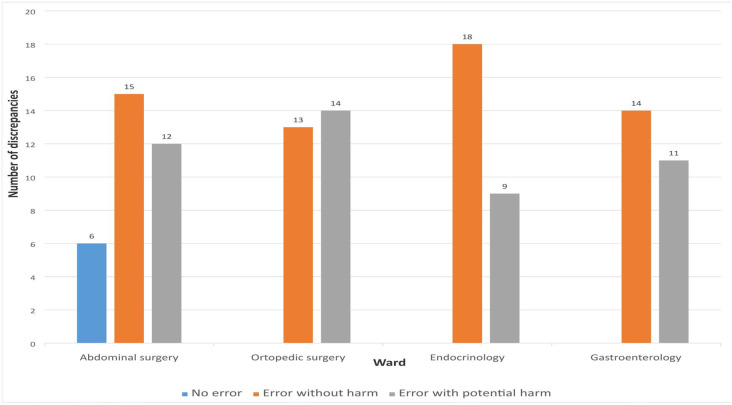
Distribution of discrepancies according to the type of medication errors.

The drug group most frequently associated with errors with potential harm was the cardiovascular system group—ATC group C (N = 37, 80.43%). The highest proportion of errors without potential to cause harm was observed with OTC medications (N = 25, 41.67%), followed by drugs from ATC group A—proton pump inhibitors (N = 14, 21.53%). Figure 5 presents the distribution of MDs according to error type.

## 4. Discussion

During the hospital admission of the 65 patients included in the study, pharmacists documented a total of 334 medications, whereas physicians recorded only 189. The number of medications documented by pharmacists was significantly higher than that recorded by physicians. These findings are consistent with previous studies, which also reported that pharmacists identified a greater number of medications at admission [4,26,27]. Specifically, in a study conducted by Abdulghani et al. involving 286 patients, physicians recorded 2548 medications, whereas pharmacists documented 3085. These findings may be attributed to the limited involvement of physicians in the medication reconciliation process at admission, possibly due to time constraints and the urgency associated with hospital admissions [26]. In our study, 145 unintentional discrepancies were identified when comparing medication data collected by pharmacists and physicians. This finding highlights the potential risk of incomplete or inaccurate medication histories when collected solely by physicians. Consistent with previous research, several factors have been associated with the occurrence of unintentional discrepancies at hospital admission, including advanced patient age, a higher number of chronic medications, and multiple comorbidities [28]. These factors may complicate the medication reconciliation process and underscore the importance of involving pharmacists to improve the accuracy and completeness of medication histories [29].

Our study also examined the association between selected factors and the number of MDs at hospital admission. A statistically significant association was observed between patient age and the number of discrepancies, indicating that older patients are more likely to experience pharmacotherapeutic inconsistencies. These findings are consistent with those reported by Guo et al., who also demonstrated a significant influence of age on the number of discrepancies (*p* < 0.001) [11]. Similarly, another study found a higher incidence of discrepancies among surgical and internal medicine patients over the age of 65 [4].

When comparing the type of hospitalization, a higher number of discrepancies was observed in patients admitted on an urgent basis; however, this difference was not statistically significant. This finding aligns with previous studies, including the study by Purat et al., which demonstrated that patients admitted emergently are at a higher risk of experiencing an increased number of MDs [9].

The most common type of unintentional MD identified at admission was omitted medication. Other types of discrepancies included incorrect dosage, incorrect dosing interval, and the documentation of medications without a corresponding indication. While previous studies have reported that the majority of patients experienced only one MD, this proportion was lower than observed in our study. The reported frequency of such discrepancies in the literature ranges from 40.8% to 83% [4,9,30,31,32].

The high percentage of omitted medications recorded by physicians may be attributed to the methods used for collecting medication histories. Physicians primarily relied on oral interviews with patients and anesthesiology reports, particularly in cases of planned surgical interventions. In contrast, pharmacists employed a more comprehensive approach, which included reviewing the patient’s medical records, conducting interviews with patients or their family members, examining medications brought to the hospital, preparing a complete and detailed list of current medications including dosing regimens, and reviewing any additional medical documentation provided by the patient. This multifaceted process likely contributed to the higher accuracy and completeness of the medication histories obtained by pharmacists.

According to the ATC classification, the drug group most frequently associated with MDs during admission was group C (cardiovascular system), followed by group A (alimentary tract and metabolism) and group N (nervous system). These findings are consistent with previous studies, which also identified cardiovascular drugs as the most frequently implicated in admission-related discrepancies. Subsequent categories commonly involved included drugs acting on the alimentary tract and metabolism (group A), the nervous system (group N), and the musculoskeletal system (group M) [4,11,23]. Guo et al. reported similar results, with group C drugs accounting for 53% of discrepancies, followed by group A (18.4%), group B (8.2%), and group N (5.1%) drugs [11]. Likewise, a 2016 study by Marinović et al. found that 40.5% of discrepancies involved cardiovascular drugs (group C), followed by group A (20.7%), group N (12.0%), and group B (9.2%) [23].

In our study, OTC medications were frequently and significantly omitted by physicians, despite their potential to influence treatment outcomes. Notably, some previous studies did not take discrepancies related to OTC medications into account [33]. However, Ling Oh et al. reported that dietary supplements, a category of OTC products, accounted for 39.0% of MDs in their study—an even higher proportion than observed in our findings [27].

In our study, the omission of cardiovascular and antidiabetic medications was classified as an error with potential to cause harm. Within the cardiovascular group, antihypertensives were the most frequently omitted subgroup, consistent with findings from previous research [23,27]. Overall, 41.07% of the MDs identified were categorized as errors with potential to cause harm.

The reported incidence of unintended discrepancies varies widely across studies, ranging from 10% to 68%. While most studies, including ours, employed the NCC MERP Pharmacotherapy Problem Categorization Index to assess the severity of discrepancies, the lack of a universally accepted standard hampers objective comparisons between studies. The proportion of discrepancies classified as errors with potential harm also differed significantly. For instance, Marinović et al. [23] reported that 60% of unintended discrepancies were potentially harmful, whereas Ling Oh et al. [27] found a much lower proportion—14.1%.

A potential explanation for the variation in our results is the inclusion of OTC medications in our analysis. In our study, the omission of OTC drugs, as well as proton pump inhibitors, was considered an error without harm. These accounted for 53.57% of all discrepancies categorized as non-harmful. For comparison, a study conducted in Saudi Arabia reported a similar proportion of non-harmful discrepancies at 48.3% [26].

This study has several limitations. First, it was conducted at a single tertiary care center across four departments, which may limit the generalizability of the findings to other hospitals or healthcare settings, particularly those with different levels of clinical pharmacy integration. Second, the study included a relatively small sample size (n = 65), which may affect the statistical power and limit the detection of smaller differences between subgroups. Third, the classification of discrepancies as intentional or unintentional relied on the judgment of pharmacists in discussion with physicians, which introduces the possibility of subjective bias. Additionally, the potential clinical impact of discrepancies was assessed using the NCC MERP Index, which, while widely used, may be interpreted differently across evaluators. Lastly, the study did not follow patients beyond admission to evaluate whether discrepancies led to actual adverse drug events or changes in clinical outcomes.

## 5. Conclusions

This study demonstrated that, upon hospital admission, clinical pharmacists documented patients’ medication therapy more comprehensively than physicians, highlighting their critical role within the multidisciplinary healthcare team. A considerable number of intentional yet undocumented changes were observed, potentially jeopardizing the continuity of care. The number of MDs was significantly associated with patient age, identifying elderly patients as a high-risk group requiring particular attention. Medication omission was the most frequent type of discrepancy, with variations observed across hospital departments. According to the NCC MERP Index, most discrepancies were classified as errors without harm, though a substantial proportion had the potential to cause harm, reinforcing the need for systematic medication reconciliation at admission.

## Figures and Tables

**Table 1:** NCC MERP Index for Categorizing Medication Errors.

No Error	Category A	Circumstances or Events That Have the Capacity to Cause Error.
Error, No Harm	Category B	An error occurred but the error did not reach the patient (an “error of omission” does reach the patient).
	Category C	An error occurred that reached the patient but did not cause patient harm.
	Category D	An error occurred that reached the patient and required monitoring to confirm that it resulted in no harm to the patient and/or required intervention to preclude harm.
Error, Harm	Category E	An error occurred that may have contributed to or resulted in temporary harm to the patient and required intervention.
	Category F	An error occurred that may have contributed to or resulted in temporary harm to the patient and required initial or prolonged hospitalization.
	Category G	An error occurred that may have contributed to or resulted in permanent patient harm.
	Category H	An error occurred that required intervention necessary to sustain life.
Error, Death	Category I	An error occurred that may have contributed to or resulted in the patient’s death.

**Table 2:** Patients’ demographic data.

Characteristic	Value
Age, years, median (IQR)	67 (58–72)
Sex, male, n (%)	20 (30.8)
BMI, kg/m^2^, mean (SD)	25.8 (±6.2)
Organizing medicines at home	
Alone, n (%)	54 (83.1)
Family, n (%)	7 (10.8)
Guardian, n, (%)	4 (6.2)
Admission to hospital	
Urgent, n (%)	35 (53.8)
Non-urgent, n (%)	30 (46.2)
Internal medicine wards, n (%)	33 (50.8)
Endocrinology, n (%)	23 (35.4)
Gastroenterology, n (%)	10 (15.4)
Surgical wards, n (%)	32 (49.2)
Abdominal surgery, n (%)	20 (30.8)
Orthopedic surgery, n (%)	12 (18.5)

IQR—interquartile range; BMI—body mass index; and SD—standard deviation.

**Table 3:** Medians in the number of recorded drugs on admission.

	Physician	Pharmacist	*p*-Value ^1^
Number of recorded drugs, median (IQR)	2 (1–4)	5 (3–7)	<0.01

^1^ Mann–Whitney *U*-test, IQR—interquartile range.

**Table 4:** Number of discrepancies in different age groups.

	<65 Years, n = 25	≥65 Years, n = 40	*p*-Value ^1^
Number of discrepancies, median (IQR)	1 (0–2.5)	2 (1–3.75)	0.035

^1^ Mann–Whitney *U*-test, IQR—interquartile range.

**Table 5:** Number of discrepancies in regard to type of admission.

	Urgent, n = 35	Scheduled, n = 30	*p*-Value ^1^
Number of discrepancies, median (IQR)	2 (1–3)	1 (0–3)	0.877

^1^ Mann–Whitney *U*-test, IQR—interquartile range.

**Table 6:** Distribution of discrepancies according to ATC classification.

ATC Class of Medicines	Medicines, n (%)
A Medicines that affect the alimentary tract and metabolism	21 (18.77)
B Medicines with effects on blood and blood organs	2 (1.75)
C Medicines that affect the cardiovascular system	37 (33.05)
G Medicines that affect genitourinary system	3 (2.69)
M Medicines that affect the musculoskeletal system	5 (4.47)
N Medicines that affect the nervous system	16 (14.29)
R Medicines that affect the respiratory system	3 (2.67)
OTC drugs	25 (22.33)

ATC—Anatomical Therapeutic Chemical, OTC—over-the-counter.

## Data Availability

The data presented in this study are available from the corresponding author upon reasonable request.

## References

[1] Sponsler K.C., Neal E.B., Kripalani S. (2015). Improving medication safety during hospital-based transitions of care. Clevel. Clin. J. Med..

[2] Abu Farha R., Yousef A., Gharaibeh L., Alkhalaileh W., Mukattash T., Alefishat E. (2021). Medication discrepancies among hospitalized patients with hypertension: Assessment of prevalence and risk factors. BMC Health Serv. Res..

[3] Lester P.E., Sahansra S., Shen M., Becker M., Islam S. (2019). Medication Reconciliation: An Educational Module. MedEdPORTAL.

[4] Mazhar F., Akram S., Al-Osaimi Y.A., Haider N. (2017). Medication reconciliation errors in a tertiary care hospital in Saudi Arabia: Admission discrepancies and risk factors. Pharm. Pract..

[5] Barnsteiner J.H. (2005). Medication Reconciliation: Transfer of medication information across settings-keeping it free from error. Am. J. Nurs..

[6] Cornish P.L., Knowles S.R., Marchesano R., Tam V., Shadowitz S., Juurlink D.N., Etchells E.E. (2005). Unintended medication discrepancies at the time of hospital admission. Arch. Intern. Med..

[7] AbuYassin B.H., Aljadhey H., Al-Sultan M., Al-Rashed S., Adam M., Bates D.W. (2011). Accuracy of the medication history at admission to hospital in Saudi Arabia. Saudi Pharm. J..

[8] Ceschi A., Noseda R., Pironi M., Lazzeri N., Eberhardt-Gianella O., Imelli S., Ghidossi S., Bruni S., Pagnamenta A., Ferrari P. (2021). Effect of Medication Reconciliation at Hospital Admission on 30-Day Returns to Hospital: A Randomized Clinical Trial. JAMA Netw. Open.

[9] Pourrat X., Corneau H., Floch S., Kuzzay M.P., Favard L., Rosset P., Hay N., Grassin J. (2013). Communication between community and hospital pharmacists: Impact on medication reconciliation at admission. Int. J. Clin. Pharm..

[10] SHPA Practice Standards (2005). SHPA Standards of Practice for Clinical Pharmacy. SHPA Committee of Specialty Practice in Clinical Pharmacy. J. Pharm. Pract. Res..

[11] Guo Q., Guo H., Song J., Yin D., Song Y., Wang S., Li X., Duan J. (2020). The role of clinical pharmacist trainees in medication reconciliation process at hospital admission. Int. J. Clin. Pharm..

[12] Dura’n-Garcı’a E., Fernandez-Llamazares C.M., Calleja-Herna’ndez M.A. (2012). Medication reconciliation: Passing phase or real need?. Int. J. Clin. Pharm..

[13] Institute for Safe Medication Practices (2025). Medicine Safety Tips: High-Alert Medicines. https://www.consumermedsafety.org/high-alert-medications.

[14] El Morabet N., Uitvlugt E.B., van den Bemt B.J.F., van den Bemt P.M.L.A., Janssen M.J.A., Karapinar-Çarkit F. (2018). Prevalence and Preventability of Drug-Related Hospital Readmissions: A Systematic Review. J. Am. Geriatr. Soc..

[15] Sarah B., Lea D., Manuel H., Adrian D., Aristomenis E., Stephan K., Evangelia L. (2021). Retrospective analysis of adverse drug reactions leading to short-term emergency hospital readmission. Swiss Med. Wkly..

[16] American Society of Health-System Pharmacists (2018). ASHP guidelines on preventing medication errors in hospitals. Am. J. Health Syst. Pharm..

[17] Doherty A.S., Shahid F., Moriarty F., Boland F., Clyne B., Dreischulte T., Fahey T., Kennelly S.P., Wallace E. (2022). Prescribing cascades in community-dwelling adults: A systematic review. Pharmacol. Res. Perspect..

[18] National Coordinating Council for Medication Error Reporting and Prevention About Medication Errors. NCC MERP Index for Categorizing Medication Errors. https://www.nccmerp.org/sites/default/files/index-color-2021-draft-change-10-2022.pdf.

[19] Urbańczyk K., Guntschnig S., Antoniadis V., Falamic S., Kovacevic T., Kurczewska-Michalak M., Miljković B., Olearova A., Sviestina I., Szucs A. (2023). Recommendations for wider adoption of clinical pharmacy in Central and Eastern Europe in order to optimise pharmacotherapy and improve patient outcomes. Front. Pharmacol..

[20] Bond C.A., Raehl C.L., Franke T. (2002). Clinical pharmacy services, hospital pharmacy staffing, and medication errors in United States hospitals. Pharmacotherapy.

[21] Shaker H.O., Sabry A.A.F., Salah A., Ragab G.M., Sedik N.A., Ali Z., Magdy D., Alkafafy A.M. (2023). The impact of clinical pharmacists’ medication reconciliation upon patients’ admission to reduce medication discrepancies in the emergency department: A prospective quasi-interventional study. Int. J. Emerg. Med..

[22] Yamada Y., Kobayashi R., Yamamoto T., Fujii H., Iihara H., Hiroko K.H., Nishida S., Hoshino R., Niwa T., Kumada K. (2024). Medication reconciliation by pharmacists for pre-admission patients improves patient safety. J. Pharm. Health Care Sci..

[23] Marinović I., Marušić S., Mucalo I., Mesarić J., Bačić Vrca V. (2016). Clinical pharmacist-led program on medication reconciliation implementation at hospital admission: Experience of a single university hospital in Croatia. Croat. Med. J..

[24] Kovacevic T., Falade J. (2025). Overcoming challenges: Implementing and scaling clinical pharmacy education and practice in the Republic of Srpska/Bosnia and Herzegovina. Int. J. Clin. Pharm..

[25] IBM Corp (2019). IBM SPSS Statistics for Windows.

[26] Abdulghani K.H., Aseeri M.A., Mahmoud A., Abulezz R. (2018). The impact of pharmacist-led medication reconciliation during admission at tertiary care hospital. Int. J. Clin. Pharm..

[27] Ling Oh A., Gerald A., Kiong Tan H., Yee Yew Chieng I. (2022). Detection of Medication Errors through Medication History Assessment during Admission at General Medical Wards. J. Pharm. Pract..

[28] Marinović I., Samardžić I., Falamić S., Bačić Vrca V. (2022). Pharmacotherapy Problems in Best Possible Medication History of Hospital Admission in the Elderly. Pharmacy.

[29] Gleason K.M., McDaniel M.R., Feinglass J., Baker D.W., Lindquist L., Liss D., Noskin G.A. (2010). Results of the Medications at Transitions and Clinical Handoffs (MATCH) study: An analysis of medication reconciliation errors and risk factors at hospital admission. J. Gen. Intern. Med..

[30] Breuker C., Macioce V., Mura T., Castet-Nicolas A., Audurier Y., Boegner C., Jalabert A., Villiet M., Avignon A., Sultan A. (2021). Medication Errors at Hospital Admission and Discharge: Risk Factors and Impact of Medication Reconciliation Process to Improve Healthcare. J. Patient Saf..

[31] Pippins J.R., Gandhi T.K., Hamann C., Ndumele C.D., Labonville S.A., Diedrichsen E.K., Carty M.G., Karson A.S., Bhan I., Coley C.M. (2008). Classifying and predicting errors of inpatient medication reconciliation. J. Gen. Intern. Med..

[32] Gellad W.F., Grenard J.L., Marcum Z.A. (2011). A systematic review of barriers to medication adherence in the elderly: Looking beyond cost and regimen complexity. Am. J. Geriatr. Pharmacother..

[33] Unroe K.T., Pfeiffenberger T., Riegelhaupt S., Jastrzembski J., Lokhnygina Y., Colón-Emeric C. (2010). Inpatient medication reconciliation at admission and discharge: A retrospective cohort study of age and other risk factors for medication discrepancies. Am. J. Geriatr. Pharmacother..

